# Fibronectin Modulates the Expression of miRNAs in Prostate Cancer Cell Lines

**DOI:** 10.3389/fvets.2022.879997

**Published:** 2022-07-11

**Authors:** Bruno Martinucci, Maira Smaniotto Cucielo, Brenda Carvalho Minatel, Sarah Santiloni Cury, Gabriel Henrique Caxali, Mirian Carolini Esgoti Aal, Sergio Luis Felisbino, Danillo Pinhal, Robson Francisco Carvalho, Flávia Karina Delella

**Affiliations:** ^1^Department of Structural and Functional Biology, Institute of Biosciences, São Paulo State University (UNESP), Botucatu, Brazil; ^2^Department of Chemical and Biological Sciences, Institute of Biosciences, São Paulo State University (UNESP), Botucatu, Brazil

**Keywords:** fibronectin, LNCaP, PC-3, RNA-seq, PI3K-AKT pathway, extracellular matrix

## Abstract

Prostate cancer (PCa) is a significant cause of cancer-related deaths among men and companion animals, such as dogs. However, despite its high mortality and incidence rates, the molecular mechanisms underlying this disease remain to be fully elucidated. Among the many factors involved in prostate carcinogenesis, the extracellular matrix (ECM) plays a crucial role. This ECM in the prostate is composed mainly of collagen fibers, reticular fibers, elastic fibers, proteoglycans and glycoproteins, such as fibronectin. Fibronectin is a glycoprotein whose dysregulation has been implicated in the development of multiple types of cancer, and it has been associated with cell migration, invasion, and metastasis. Furthermore, our research group has previously shown that fibronectin induces transcriptional changes by modulating the expression of protein coding genes in LNCaP cells. However, potential changes at the post-transcriptional level are still not well understood. This study investigated the impact of exposure to fibronectin on the expression of a key class of regulatory RNAs, the microRNAs (miRNAs), in prostate cancer cell lines LNCaP and PC-3. Five mammalian miRNAs (miR-21, miR-29b, miR-125b, miR-221, and miR-222) were differentially expressed after fibronectin exposure in prostate cell lines. The expression profile of hundreds of mRNAs predicted to be targeted by these miRNAs was analyzed using publicly available RNA-Sequencing data (GSE64025, GSE68645, GSE29155). Also, protein-protein interaction networks and enrichment analysis were performed to gain insights into miRNA biological functions. Altogether, these functional analyzes revealed that fibronectin exposure impacts the expression of miRNAs potentially involved in PCa causing changes in critical signaling pathways such as PI3K-AKT, and response to cell division, death, proliferation, and migration. The relationship here demonstrated between fibronectin exposure and altered miRNA expression improves the comprehension of PCa in both men and other animals, such as dogs, which naturally develop prostate cancer.

## Introduction

Prostate cancer (PCa) remains the most common malignancy and a major cause of cancer-related deaths among men (1). Several aspects of prostate cancer are similar in men and other mammals like dogs that often exhibit spontaneous development and poor prognosis (2, 3). During the development of this disorder, several paracrine stimuli between acinar epithelial cells and the surrounding environment modulate cell behavior and the expression of stromal extracellular matrix (ECM) molecules (4, 5).

Among the components of the ECM, fibronectin (FN) plays a prominent role, regulating a wide spectrum of cellular events (6, 7). This glycoprotein has been implicated in the development of multiple types of cancer (8–10). FN confers cell advantages as a scaffold for cell invasion and the growth of new blood vessels, as well as increases cell proliferation (10–12). Our research group has previously shown that LNCaP cells, a neoplastic prostate cell line, display an altered expression of genes involved in cell adhesion, survival, and proliferation when exposed to FN (13).

The ECM composition and behavior are regulated at multiple levels in prostate cancer cells, and microRNAs (miRNAs) are crucial components of this regulation at the post-transcriptional level. MiRNAs are small non-coding RNAs with ~ 17 to 25 nucleotides that mediate gene silencing through target mRNA cleavage and degradation, or translational repression (14–16). Due to their ability to target several mRNAs, miRNAs can coordinate or fine-tune the expression of many proteins. Given that a significant fraction of miRNAs appears to be conserved over long evolutionary times, they are valuable molecules for cross-species functional inferences. In mammals, predictions indicate that miRNAs control the activity of ~50% of all protein-coding genes (17), promoting an orchestrated regulation of signaling pathways and complex biological functions (18, 19).

However, while it has been well established that miRNAs can regulate the expression of ECM molecules, just recently, it has become apparent that the expression and function of miRNAs can be influenced by ECM components (20, 21). Understanding how ECM components such as FN control the expression of miRNAs may facilitate the discovery of molecular mechanisms underlying PCa progression and unveil new therapeutic opportunities.

In this study, differentially expressed miRNAs were identified in both prostate cell lines LNCaP (androgen-sensitive) (22) and PC-3 (androgen-independent) (23) after FN exposure. Next, online databases were searched for validated mRNA targets of these differentially expressed miRNAs and their expression patterns were also explored. Finally, these mRNA targets were annotated in protein-protein interaction networks and functional enrichment analysis was performed to gain further insights into the biological contexts they are enrolled in.

## Materials and Methods

### Cell Lines and Culture Conditions

Prostate cancer cell lines LNCaP (clone FGC - ATCC^®^ CRL-1740™) and PC-3 (ATCC^®^ CRL-1435™) were obtained from the American Type Culture Collection (ATCC). Both cell lines were cultured in RPMI 1640 medium (Gibco BRL, Grand Island, NY) containing 10% fetal bovine serum (FBS - Gibco BRL, Grand Island, NY) and 1% 100 × Antibiotic/Antimycotic (Gibco BRL, Grand Island, NY). Cells were maintained in a humidified incubator with 5% CO_2_ at 37°C.

### Fibronectin Exposure

Cells were seeded at 4 × 10^4^ cells/cm^2^ and exposed to human plasma derived FN (Sigma-Aldrich; F0895) at a concentration of 25 μg/ml, according to a protocol previously established (13, 24). The experiments were carried out in triplicates.

### RNA Extraction

RNA was isolated from LNCaP and PC-3 cells using the PureLink^®^ RNA kit (Invitrogen, Carlsbad, CA) according to the manufacturer's instructions. The quantification and quality of the RNA were checked by NanoVue™ Plus spectrophotometer (GE HealthCare, Little Chalfont, UK) and Agilent 2100 Bioanalyzer (Agilent Technologies, Palo Alto, CA) (25), respectively. Only samples with RNA integrity numbers above nine were used in further analysis. The DNAse I, Amplification Grade (Invitrogen, Carlsbad, CA) was used to remove genomic DNA.

### miRNAs Expression by RT-qPCR

The expression of seven miRNAs known to be associated with PCa was evaluated (Table 1).

**Table 1:** MicroRNAs evaluated after fibronectin exposure in prostate cancer cells.

**miRBase ID**	Assay ID^*^	**Reference**
hsa-miR-21-5p	000397	(26)
hsa-miR-29b-3p	000413	(27)
hsa-miR-34a-5p	000425	(28)
hsa-miR-125b-5p	000449	(29)
hsa-miR-145-5p	002278	(30)
hsa-miR-221-3p	000524	(31)
hsa-miR-222-3p	002276	(19)

* *Taqman® Assay ID*.

For this, 10 ng of total RNA was reverse transcribed using TaqMan MicroRNA Reverse Transcription Kit and miRNA-specific primers (Thermo Fisher Scientific Inc., Waltham, MA, USA). The reaction conditions were as follows: 16°C for 30 min, 42°C for 30 min, and 85°C for 5 min. The qPCR reactions were performed on a QuantStudio™ 12K Flex system (Thermo Fisher Scientific Inc., Waltham, MA, USA) using TaqMan^®^ Fast Advanced Master Mix and sequence-specific TaqMan MicroRNA Assays (Table 1/Thermo Fisher Scientific Inc., Waltham, MA, USA). Relative gene expression was calculated using the 2^−Δ*ΔCt*^ method (32), and levels were normalized using the endogenous reference RNAs: RNU43 and RNU48. Statistical analyses were performed using parametric one-way ANOVA test with the “a posteriori” Tuckey–Kramer test. Differences were considered statistically significant when *p* < 0.05.

### Identification of Target Genes for the Differentially Expressed miRNAs

Each miRNA commonly has a plurality of target genes. To identify the possible target genes of the differentially expressed miRNAs, miRTarBase v.9.0 (33) database was employed. These two databases use different algorithms to predict experimentally validated miRNA targets. It is important to mention that only interactions proven by the following techniques were retained for further analysis: (i) reporter assay; (ii) Western blot; and (iii) RT-qPCR.

### Gene Expression Profile in LNCaP and PC-3 Cells

To evaluate the expression levels of the validated mRNA targets in LNCaP and PC-3 cells, the mRNA expression profiles of both cell lines available at The Gene Expression Omnibus (GEO) database (www.ncbi.nlm.nih.gov/geo/) were downloaded. The accession number of the LNCaP mRNA expression profile is GSE29155 (34), containing seven samples from LNCaP cells and four samples of prostate epithelial cells (PrEC), which were used as a control in this study. The mRNA profiles were detected using the platforms GPL9052 (Illumina Genome Analyzer) and GPL9115 (Illumina Genome Analyzer II). The accession numbers of PC-3 mRNA expression profiles are 8GSE64025 (35) and GSE68645 (36), and both contain two samples for PC-3 cells. The platform used was GPL10999 (Illumina Genome Analyzer IIx) in GSE64025 and GPL16791 (Illumina Hiseq 2500) in GSE68645.

The RNA-sequencing analysis for all samples was performed using the Galaxy platform (www.usegalaxy.org) (37). After uploading the raw expression data obtained from GEO, a FASTQc analysis (version 0.11.8) was performed to check for quality of runs. Subsequently, adapter removal and quality filtering of the samples were performed using the Trimmomatic software (version 0.38) (38). The following parameters were used as filters: LEADING:3 TRAILING:3 SLIDINGWINDOW:4:15 and MINLEN:22. Next, alignment was performed with the genome of *Homo sapiens*, version hg19, using the HISAT2 tool (version 2.2.1) (39). The FeatureCounts tool (40) was used to obtain the counts. Finally, the data in counts was used in DESeq2 (41) to obtain the differentially expressed genes (DEGs; FC: 1.2 and *p*_adj_ < 0.05). In this last analysis, data from PrEc cells were used as a comparative control against cancer cell lines data.

### Construction of Interaction Networks for the miRNA Target Genes

To verify the synergy among proteins associated with DEGs regulated by the selected miRNAs, protein-protein interaction (PPI) networks were evaluated using the online software STRING v.11.5 (42) (http://string-db.org). The following parameters established by de Oliveira et al. (43) were used for the construction of these networks: Meaning of network edges: confidence; Active interaction sources: Experiments, database, co-expression, neighborhood, co-occurrence; Minimum required interaction score: high confidence of 0.900; Display simplifications: hide disconnected nodes in the network (43).

### Functional Enrichment Analysis

The set of differentially expressed genes found to be targets of the selected miRNAs was submitted to the EnrichR tool (https://maayanlab.cloud/Enrichr/) (44). The following libraries were selected: “Biological Process 2021” from Gene Ontology (GO) and KEGG 2021. Data were ranked according to the combined score and classified according to the percentage of enriched genes per term and the –Log_10_ *p*-value.

## Results

### Impact of Fibronectin Exposure on miRNA Expression

**Figure 1:**
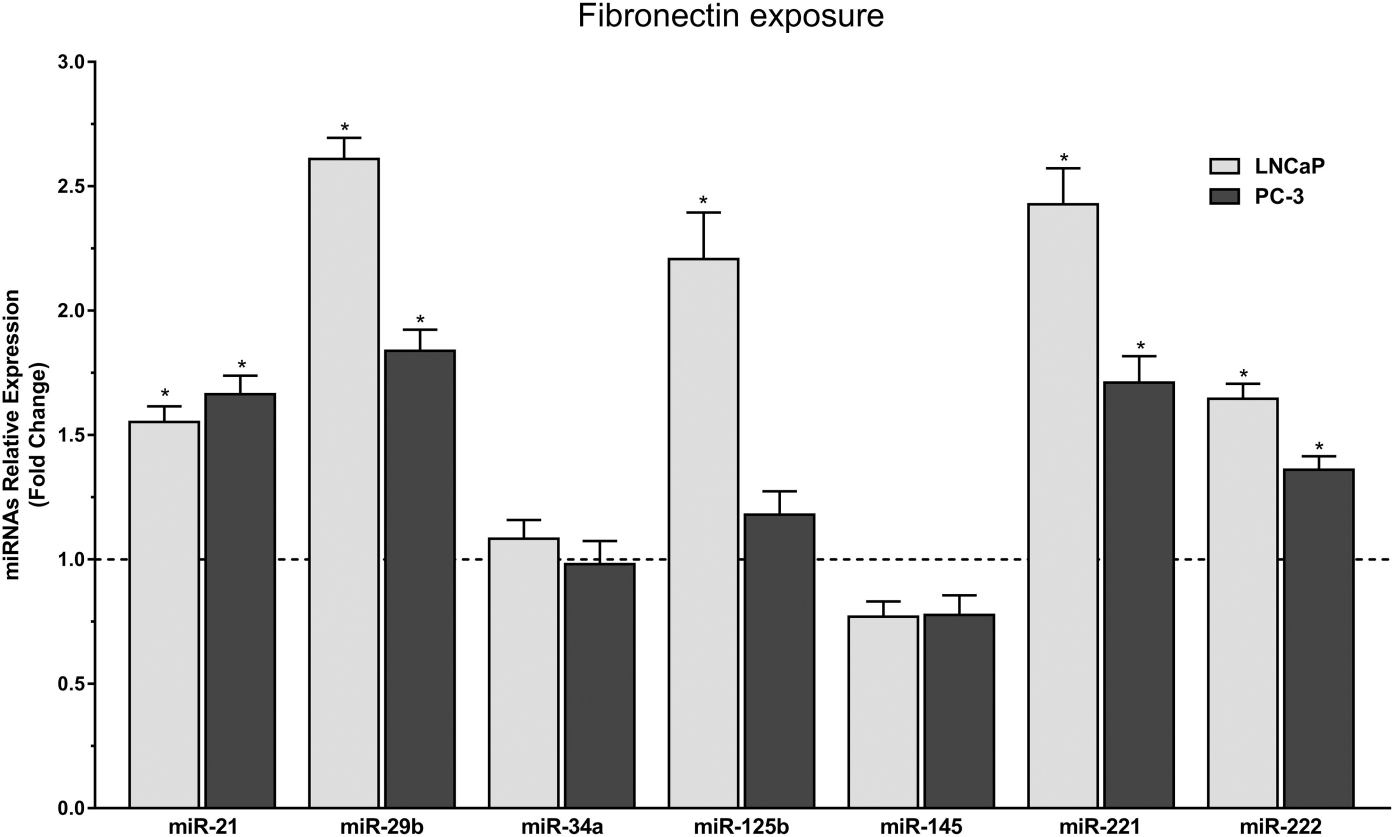
Relative expression of miRNAs after FN exposure in LNCaP and PC-3 cells (Fold Change from unexposed cells from each cell line). Light grey bars represent the miRNA levels of LNCaP cells exposed to FN, and dark grey bars represent the PC-3 cells. The line represents the value of non-exposed LNCaP and PC-3 cells. Values are expressed by mean ± SEM (Standard error of the mean *p < 0.05 between cells exposed and non-exposed in the same cell line).

In order to better understand the impact of FN on the molecular mechanisms of PCa, the expression of seven miRNAs known to be involved in this disease were evaluated by RT-qPCR in both LNCaP and PC-3 cell lines (Figure 1). LNCaP cells exposed to FN displayed increased expression levels of the miRNAs hsa-miR-21-5p (1.55 fold), hsa-miR-29b-3p (2.61 fold), hsa-miR-125b-5p (2.20 fold), hsa-miR-221-3p (2.42 fold), and hsa-miR-222-3p (1.64 fold) when compared to non-exposed LNCaP cells. In PC-3 cells, with the exception of hsa-miR-125b-5p, all other miRNAs differentially expressed in LNCaP also showed a significant increase in expression, as observed for hsa-miR-21-5p (1.66 fold), hsa-miR-29b-3p (1.83 fold), hsa-miR-221-3p (1.71 fold), and hsa-miR-222-3p (1.36 fold). In PC-3, smaller differences in relative expression between unexposed and exposed cells were observed. Two miRNAs, hsa-miR-34a-5p and hsa-miR-145-5p, had similar but non-significant changes in relative expression levels in both cell lines and culture conditions.

### Target Genes of the Differentially Expressed miRNAs

**Figure 2:**
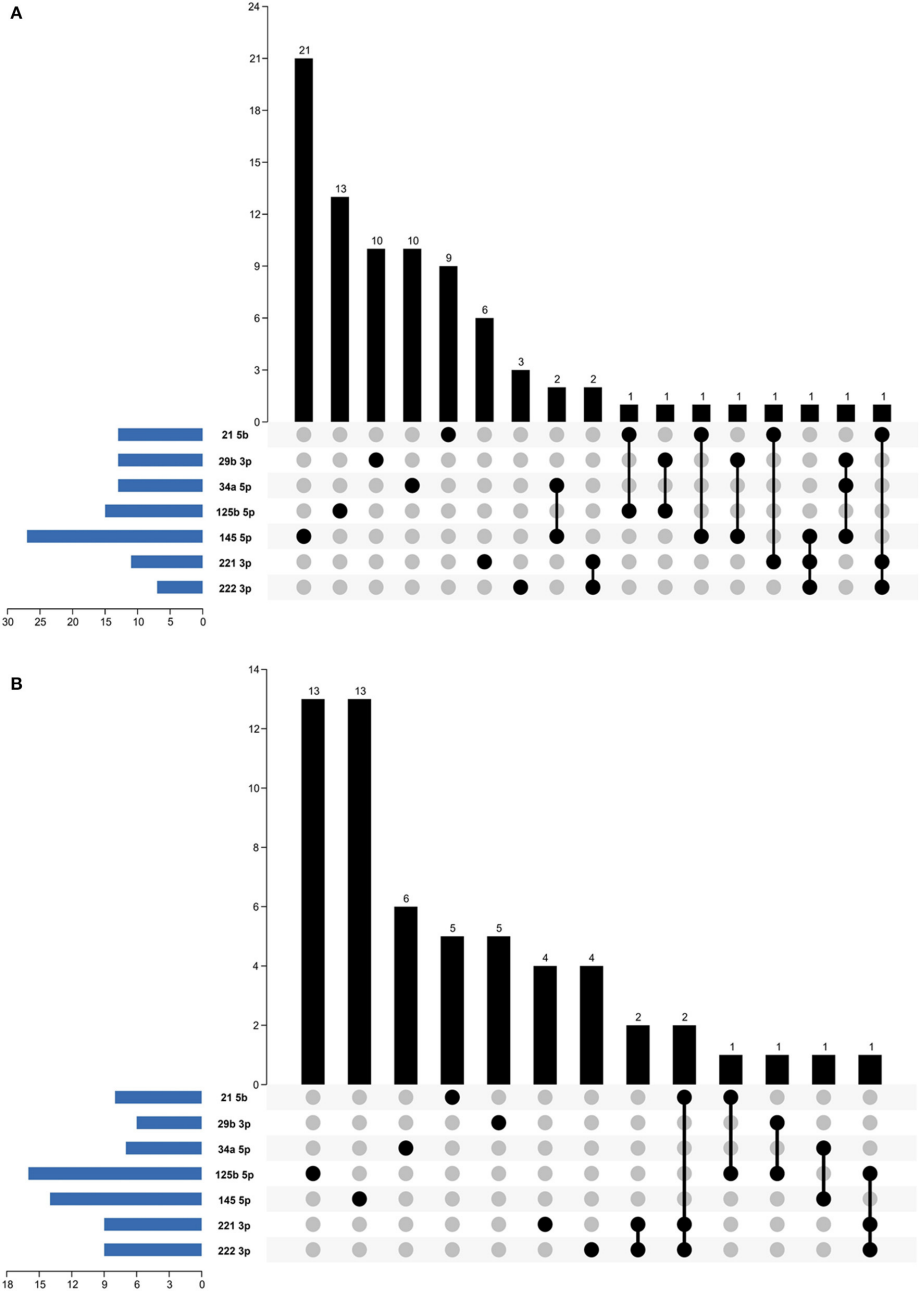
Gene distributions among selected miRNAs. The band in the lower-left corner shows the number of genes targeted by miRNA. The dot and line in the lower right corner represent the subsets of gene events shared between the miRNA targets. The number of relevant genes in each subset is represented in the histogram, which is the upper part of the graph. **(A)** LNCaP; **(B)** PC-3.

To explore the biological functions of the five differentially expressed miRNAs, their validated mRNA targets were obtained from two databases and compiled (Figure 2 and Table 2). A total of 141 targets were recovered, with little overlapping among the subset of targets from every differentially expressed miRNA within each PCa cell lineage (Figure 2). This means that most of targets used in the downstream analysis have a single mRNA as target. A maximum of six subsets of gene events were shared between the miRNA targets in both lineages. For instance, miR-221-3p have in common with other differentially expressed miRNAs five distinct targets, of which three were also targeted by miR-222-3p. By contrast, miR-125b-5p had only two targets shared in LNCaP cells (Figure 2A) and miR-29b-3p had a single target in common with another miRNA in PC-3 cells (Figure 2B).

**Table 2:** Target genes of differentially expressed miRNAs.

**(A) LNCaP**				
**hsa-miR- 21-5p**	**hsa-miR- 29b-3p**	**hsa-miR- 125b-5p**	**hsa-miR- 221-3p**	**hsa-miR- 222-3p**
APAF1	CCND2	BAK1	ADAMTS6	ESR1
BTG2	CDC42	BTG2	ANXA1	ETS1
DUSP10	CDK6	CYP24A1	APAF1	FOS
HPGD	COL3A1	DUSP6	ARF4	GNAI3
IL12A	COL4A1	EIF5A2	DDIT4	MMP1
MSH2	DNMT3B	FGFR2	ESR1	SOD2
MTAP	GRN	IKZF2	ETS1	TIMP3
SERPINB5	HMGA2	LIPA	FOS	
SPRY2	ITGA6	MCL1	RAB1A	
TGFBR2	LAMC2	MXD1	RB1	
TIMP3	MCL1	PCTP	TIMP3	
TP63	MMP2	PRDM1		
TPM1	PER1	RPS6KA1		
		TNFAIP3		
		VDR		
**(B) PC-3**				
**hsa-miR- 21-5p**	**hsa-miR- 29b-3p**	**hsa-miR- 125b-5p**	**hsa-miR- 221-3p**	**hsa-miR- 222-3p**
BTG2	CCND2	BBC3	ADAMTS6	BBC3
DUSP10	ITGA6	BCL2L2	ANXA1	ESR1
PTEN	LAMC2	BTG2	BBC3	FOS
SERPINB5	MCL1	CYP24A1	DDIT4	GAS5
SMAD7	MMP2	DUSP6	ESR1	MMP1
TIMP3	PER1	ERBB2	FOS	PPP2R2A
TP63		ERBB3	MDM2	PTEN
TPM1		FGFR2	PTEN	STAT5A
		IKZF2	TIMP3	TIMP3
		MCL1		
		MXD1		
		PRDM1		
		SCNN1A		
		TP53		
		TP53INP1		
		VDR		

### Protein-Protein Interactions (PPI) Networks for Target DEGs

**Figure 3:**
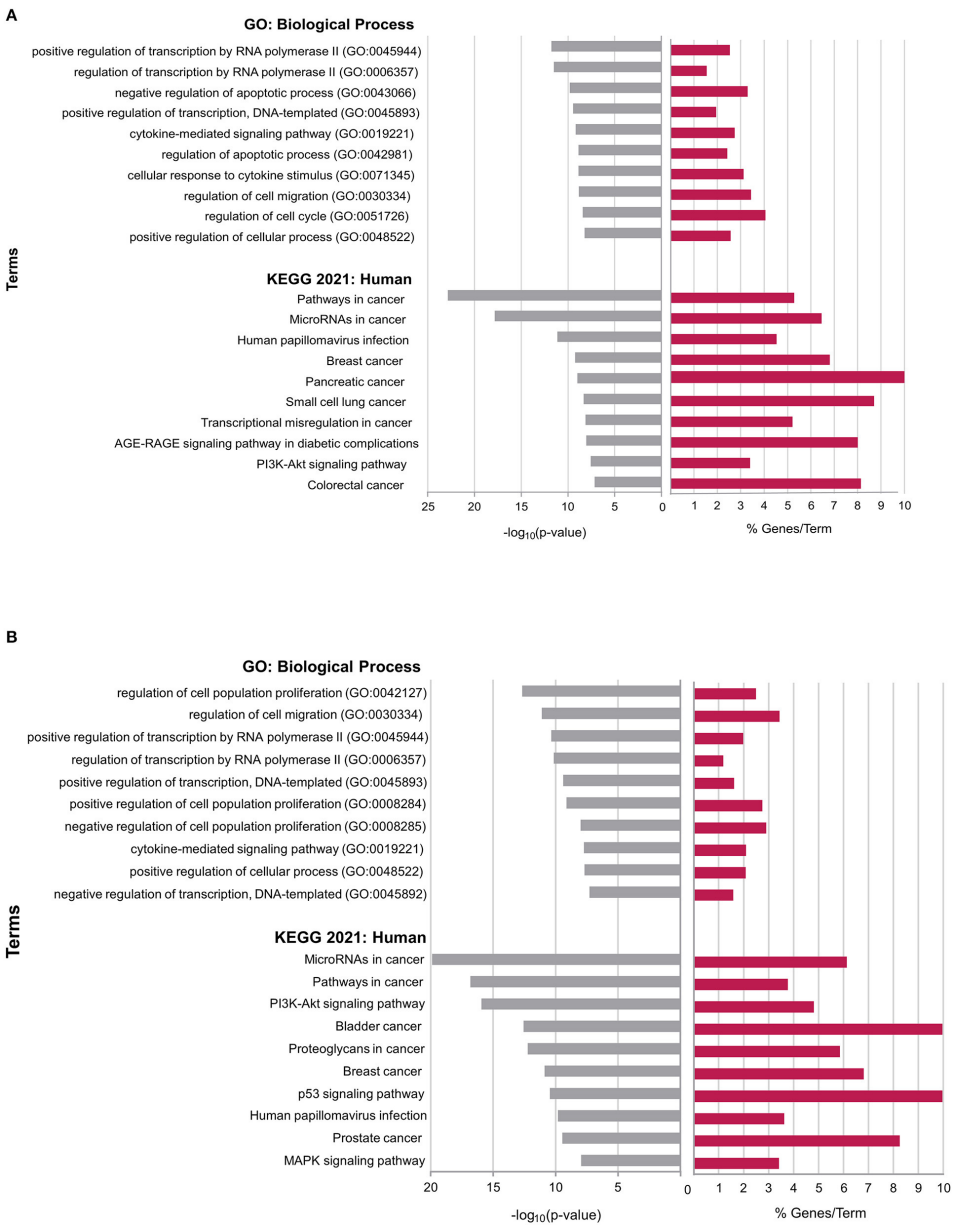
**(A,B)** Functional classification of upregulated genes that are miRNA targets evaluated in LNCaP and PC-3 cells according to the EnrichR tool.

The online software STRING was used to predict PPIs networks of DEGs in LNCaP and PC-3 cells (Figure 3). The combined scores that weigh the degree of confidence for each interaction are illustrated in Supplementary Table 2. The network was constructed with 84 target genes in LNCaP cells and 57 target genes in PC-3 cells. In the LNCaP network, FOS, MCL1, BAK1, APAF and CDC42 appeared as key nodes (red circles), while TP53, MCL1, BBC3, MDM2, and PTEN appeared as key nodes (red circles) in the PC-3 network.

### Functional Enrichment Analysis

**Figure 4:**
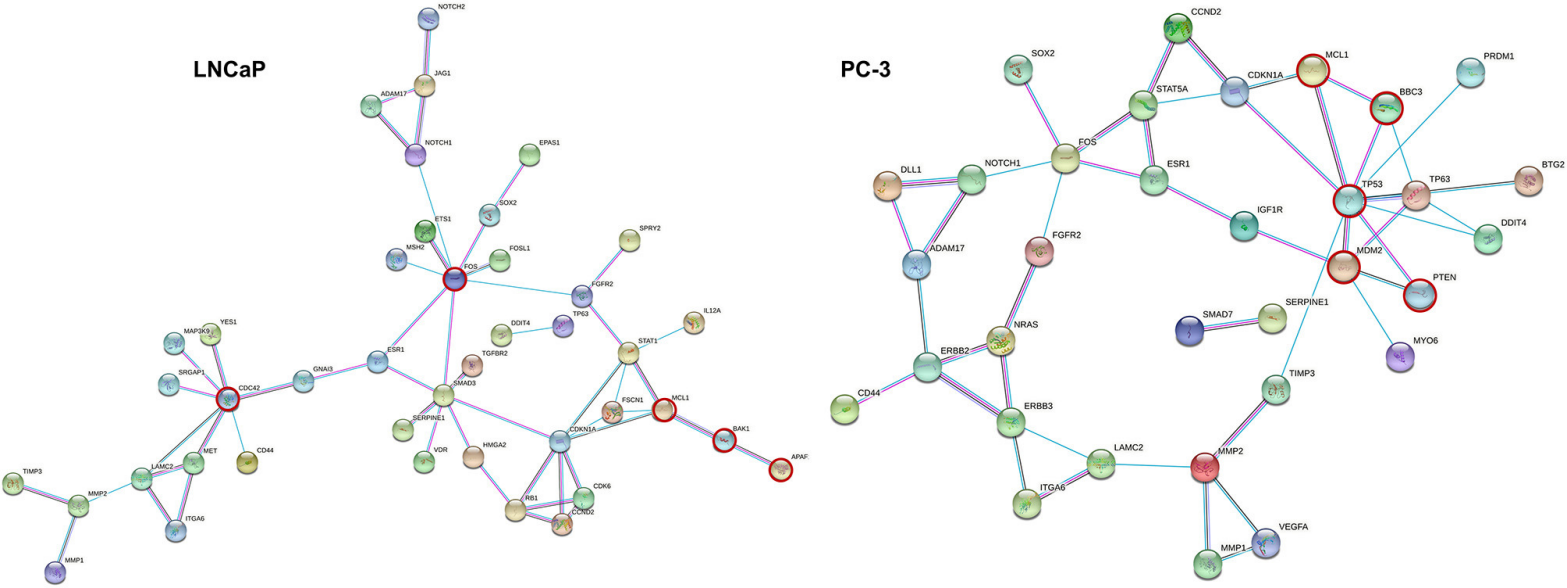
Interaction network of upregulated DEGs generated by STRING software in LNCaP and PC-3 cells. Circles represent genes, and lines represent protein-protein associations. Edges represent protein-protein associations; known Interactions: from curated databases (light blue), experimentally determined (fuchsia); co-expression (black) and protein homology (light cyan).

To perform the functional enrichment analysis of the DEGs targeted by the selected miRNAs, ontology (GO Biological Process 2021) and pathway (KEGG 2021, Human) databases were assessed, as shown in Figure 4. LnCaP cell lines are enriched mainly for ontology terms related to the regulation of transcription or cell division and death. The pathways are related to several types of cancer (breast, pancreatic, small cell lung, and colorectal), in addition to miRNA regulation in cancer, with an emphasis on the PI3K-AKT pathway, which was the only one specified in the enrichment.

The PC-3 cell line presented similar results to that seen in LnCaP, with many processes related to transcriptional regulation and a range of processes associated with tumor progression, including the regulation of cell proliferation and migration. This cell line also presented pathways related to miRNA regulation in cancer and important pathways such as PI3K-AKT, p53, and MAPK.

## Discussion

In the present study, we analyzed the impact of FN on the expression of miRNAs in the neoplastic prostate cell lines LNCaP and PC-3, which are widely employed in PCa research. The seven miRNAs examined regulate genes that are known to display altered expression in response to FN exposure (13).

### FN Stimulates a Differential Expression of Critical PCa-Related miRNAs

Our results have demonstrated that FN exposure is capable of altering the expression of miRNAs in both prostate cell lines. LNCaP and PC-3 demonstrated higher expression levels of miR-21, miR-29b, miR-221, and miR-222. In addition, LNCaP cells also displayed an increased expression of miR-125b.

Previously, miR-21 was shown to be upregulated in PCa (26). In fact, miR-21 is related to the development of many types of human cancers, functioning as an oncogene by targeting tumor suppressor genes, such as PTEN, in cancer cells (45).

MiR-29b is expressed at low levels in prostatic tumor tissues (27) and, when upregulated, inhibits Mcl-1, matrix metalloproteinase-2 (MMP-2), and collagen. Similarly, other studies show that miR-29b is under-expressed in several types of cancer, in instances where it works as a tumor suppressor. Conversely, there are some studies reporting oncogenic functions for miR-29b that should be considered in future investigations (46).

Among the most crucial miRNA families, the miR-125 family has been reported to be involved in a variety of carcinomas (47). miR-125b targets multiple genes involved in the regulation of apoptosis, including *BAK1* and *STAT3*, but depending on the cell type, miR-125b can either contribute to oncogenesis or tumor suppression (29, 48). In PCa, this miRNA was upregulated and could be responsible for stimulating the androgen-independent growth (29), which may help to explain the increased expression of miR-125b restricted to LNCaP cells after FN exposure, once PC-3 cells are already insensitive to androgen.

It is well-known that overexpression of the miR-221 and miR-222 promotes growth, metastasis, and invasion of a variety of malignant tumors, including PCa (49). In addition, these two miRNAs were observed to affect the proliferative potential of human prostate carcinoma cells (31). Kobayashi et al. (50) investigated the expression of several miRNAs in canine non-tumor prostatic tissue and prostatic adenocarcinoma tissue, demonstrating that miR-221 and miR-222 were significantly upregulated in prostatic adenocarcinoma tissue. Besides morphological and functional similarities between human and dog prostate (2), the differential expression of miRNAs can also be similar in both. Thus, further studies are required to verify the influence of ECM, especially FN, on the expression of these molecules in canine prostate cancer.

### Protein-Protein Interactions (PPI) Networks and Pathway Analysis

Given the importance of the investigated miRNAs in PCa, we sought to understand how their mRNA targets would interact in the evaluated cell lines. For this, miRNA target information was retrieved from miRTarBase and the expression levels of these mRNAs were evaluated in publicly available expression profile data of LNCaP and PC-3 cells. Finally, gene ontology, pathway enrichment, and protein interaction analysis of these candidate genes were performed to better determine the putative effects of FN regulation in PCa. Among the several mRNA targets of the five miRNAs scrutinized, 84 and 57 genes were differentially expressed in the LNCaP and PC-3 cell lines, respectively. Therefore, these were chosen for further PPI analysis.

The protein products of the selected target genes were submitted to the STRING platform. Based on the PPI network analysis presented here, it was possible to highlight the importance of some genes for the regulation of basic biological processes that maintain tumor progression.

For instance, miR-125b overexpression after FN exposure can contribute to the progression of PCa, since this miRNA negatively regulates TP53 protein expression. The *TP53* gene, a tumor suppressor that acts as a negative regulator of *MDM2*, establishes many relationships in the network formed for the PC-3 cell line. *MDM2* is an oncogene related to several human cancers (51, 52) and canine neoplasms (53, 54). Furthermore, its expression is associated with metastatic and recurrent cancers (55), by promoting inhibition of TP53 activity and altering cell cycle control, DNA repair, among others (56–58). In the case of PCa, Tian et al. (59) evaluated HGC-27 cells transfected with miR-509-5p, a miRNA that downregulated MDM2, and observed a decrease in migration/invasion and inhibition of cell proliferation. Furthermore, PC-3 cells treated with the MDM2-TP53 interaction inhibitor showed an increase in apoptosis rates. Therefore, blocking MDM2 may be a promising target for PCa treatment (60). This result demonstrates the crucial role that TP53 protein plays in the progression of several tumors, since the expression of *TP53* gene is altered in more than 50% of tumors (51, 52).

The PPI results also showed that PTEN has significant interactions in PC-3 cells. PTEN is a well-known tumor suppressor that is frequently dysregulated throughout the course of cancer. The activation of the PI3K signaling pathway, which is recognized as one of the most common routes observed throughout the tumor growth process in numerous types of malignancies, including Pca, is caused by the loss of function of PTEN (61).

Activation of the PI3K/AKT pathway clearly plays a major role in the aggressive nature of PCa (62). Studies demonstrate that an improved understanding of the biology of this pathway in Pca will be important to better comprehend the benefits from PI3K/AKT inhibitors and which point in the disease course these inhibitors should be given (63). In our study, PTEN was found to target by three miRNAs (i.e., miR-21, miR-221 and miR-222) that were overexpressed after exposition to FN. In addition, other differentially expressed target genes were found to be involved in the PI3K/AKT signaling pathway. Thus, we believe that FN modulatory effect over miRNA expression could be a key factor in the regulation of the PI3K/AKT pathway, thereby contributing in the development of the resistant form of Pca.

Another molecule that showed outstanding enrichment in both PC-3 and LNCaP lineages and that was related to TP53 in the PPI was the anti-apoptotic factor MCL-1, a member of the BCL-2 family, and a predicted target of miR-29b, tumor suppressor, and interestingly is also target of miR-125b. MCL-1 has been shown to confer advantages to Pca cells through the inhibition of BAX and BAK, two pro-apoptotic proteins, as well as and blockage of cytochrome -c release (64). The interaction of BAK, target of miR-125b, with MCL-1 was also highlighted in the network formed from the up-regulated genes of the LNCaP lineage. In castration-resistant and androgen-independent Pca, as in the case of PC-3 cells, MCL-1 is upregulated, resulting in resistance to apoptosis and a worse prognosis (65). Other authors have showed increased apoptosis in PC-3 cells after treatment with thymoquinone, an agent capable of negatively regulating MCL-1 (66). Other studies also demonstrate higher cell death rates in PC-3 cells after treatment with drugs that inhibit MCL-1 (67, 68).

BBC-3, also known as PUMA, is a positive regulator of apoptosis that is directly related to the BCL-2 family (69). In a study using colorectal cancer cells, BBC-3-transcriptional activation was seen to mediate TP53-associated cell death (70). Here BBC-3 formed an interaction network with MCL-1 and TP53, which have already had their anti- and pro-tumor activities discussed above. BBC-3 was found as a predicted target of the miR-125b, miR-221 and miR-222, all differentially expressed by FN exposition. These miRNAs are known for their pro-tumor activity, down-regulating antitumor molecules (71).

Within the LNCaP lineage PPI network, besides MCL1, BAK1 (above discussed) we call attention to FOS and APAF. Although members of the FOS family (predicted as targets of miR-221 and miR-222) are generally associated with oncogenesis, early recurrence, and increased survival of prostatic tumor cells (72, 73), there is no consensus on its role. Authors such as Mahner et al. (74) found they are related to a higher survival rate among ovarian cancer patients, and showed c-FOS to play antiangiogenic and pro-apoptotic roles in several types of cancer, although the exact mechanisms by which c-FOS contributes to apoptosis are unclear.

APAF1 (apoptotic protease activating factor), has been named as a critical regulator of TP53-dependent apoptosis since it is a direct transcriptional target of the tumor suppressor TP53 (75). In primary murine fibroblasts, loss of APAF-1 activity enhances transformation and chemoresistance, which is consistent with TP53 loss (76). In our study, this protein was seen as a predicted target of miR-21 and miR-221, both oncomiRs. This observation suggests a protumoral role of FN on the observed miRNAs.

Another protein that was central in several interactions detected in the PPI analysis was CDC42, which is part of the family of Rho GTPases (77). CDC42 is a predict target of miR-29b. This protein acts in several fundamental processes for tumor progression, such as cell division, enzymatic activity, cell polarity, invasion, and transformation (78). Furthermore, CDC42 has already been described as a critical mRNA target for miRNAs in the progression of PCa, through *in silico* analysis, using tools similar to those used in the present study (79). Among the effects of CDC42 on PCa tumorigenesis, it was demonstrated association with ACK1 kinase and increased disease progression. CDC42 seems to promote the recruitment of androgen receptors, causing increased cell invasion and metastasis, thus leading to the rapid development of xenographic tumors in mice (77). This increase in invasion and cell migration is because CDC42 acts in the modulation of cytoskeleton plasticity, where its higher levels contribute to the modulation of an invasive cell phenotype in PCa (80). In this way, CDC42 is an essential molecule for PCa tumorigenesis and can be targeted for potential treatments. Thus, in contrast to mostly negative effects, FN can be beneficial to cells by raising the expression of miR-29b, which has the potential to dampen CDC42 tumorigenic activity.

In summary, few studies have investigated how ECM can modulate miRNA expression, and here, we investigated the impact of FN exposure on miRNA expression in LNCaP and PC-3 cells. Five miRNAs were found differentially expressed and possibly are involved in the progression of PCa through the modulation of signaling pathways such as PI3K-AKT and pathways related to cell division, death, proliferation, and migration. Therefore, a better understanding of the relationship between FN and miRNA expression can improve the understanding of PCa progression. In addition, the subset of conserved mammalian miRNAs here examined may be of great relevance to improve the efficacy of PCa therapies.

## Data Availability Statement

The datasets presented in this study can be found in online repositories. The names of the repository/repositories and accession number(s) can be found in the article/Supplementary Material.

## Author Contributions

BM, MC, and BCM developed and analyzed the *in vitro* experiments. In addition, they planned and performed the bioinformatics analyzes together with SC, GC, and MA. FD, SF, RC, and DP conceived the project and helped with data analysis and interpretation. FD was the general coordinator of the research project. All authors contributed to the article and approved the submitted version.

## Funding

This article comprises part of the Master's dissertation of BM, supported by FAPESP funding (grant #2014/25702-0 and #2013/26114-2). This study was financed in part by the Coordenação de Aperfeiçoamento de Pessoal de Nível Superior - Brasil (CAPES) - Finance Code 001.

## Conflict of Interest

The authors declare that the research was conducted in the absence of any commercial or financial relationships that could be construed as a potential conflict of interest.

## Publisher's Note

All claims expressed in this article are solely those of the authors and do not necessarily represent those of their affiliated organizations, or those of the publisher, the editors and the reviewers. Any product that may be evaluated in this article, or claim that may be made by its manufacturer, is not guaranteed or endorsed by the publisher.

## Supplementary Material

Supplementary Table 1 | Complete list of differentially expressed genes in LNCaP and PC-3 cells.

Supplementary Table 2 | STRING Interactions in LNCaP and PC-3 cells.
