# Supplementary material for: Fibronectin Modulates the Expression of miRNAs in Prostate Cancer Cell Lines
Source: Front Vet Sci. 2022 Jul 11;9:879997. doi: 10.3389/fvets.2022.879997 (PMC9310065; doi:10.3389/fvets.2022.879997)
Supplement: Supplementary Table 1 | — Complete list of differentially expressed genes in LNCaP and PC-3 cells. [file Table_1.pdf]

Differentially expressed genes in both cell lines							
LnCaP				*PC-3*			
**Gene**	**log2(FC)**	**P-value**	**P-adj**	**Gene**	**log2(FC)**	**P-value**	**P-adj**
ITGA6	4.585857	2.7E-263	3.5E-260	CCND2	10.94613	1.5E-106	6.2E-103
SERPINB5	8.391191	7.2E-240	6.7E-237	MMP2	8.969008	6.19E-61	3.47E-58
KLF5	6.054233	4E-128	8.8E-126	SCNN1A	7.517658	8.3E-57	3.14E-54
EPAS1	6.113061	2.1E-115	3.4E-113	TP53	5.212684	4.13E-49	1.05E-46
LAMC2	15.81223	4.48E-97	5.01E-95	TP63	9.498666	2.47E-47	5.65E-45
JAG1	2.196641	7.86E-88	7.21E-86	BTG2	6.433306	2.61E-43	4.56E-41
CD44	12.33859	2.18E-87	1.99E-85	TIMP3	8.703089	1.29E-35	1.53E-33
CDK6	7.22499	6.33E-82	5.02E-80	FGFR2	7.041571	9.25E-28	6.4E-26
DUSP6	8.231397	1.63E-80	1.24E-78	PTEN	3.867297	1.89E-26	1.18E-24
ETS1	7.564686	9E-78	6.42E-76	ANXA1	2.457878	4.31E-25	2.42E-23
MICAL2	2.727697	1.22E-74	8.19E-73	LAMC2	3.923802	3.31E-17	1.03E-15
SMAD3	3.354942	1.75E-70	1.06E-68	FOS	5.284813	4.91E-16	1.39E-14
ADAM17	2.888609	1.59E-67	9.15E-66	CD44	2.3571	6.11E-16	1.72E-14
ANXA1	12.38917	5.76E-61	2.72E-59	SERPINB5	4.343776	1.24E-15	3.43E-14
TGFBR2	9.824538	3.28E-59	1.49E-57	SOX2	6.685487	5.69E-14	1.28E-12
SERPINE1	12.64382	4.21E-59	1.9E-57	TP53INP1	2.151772	5.89E-14	1.32E-12
CCND2	11.9748	9.32E-58	4.09E-56	ITGB8	2.573166	1.39E-12	2.67E-11
CDKN1A	1.811404	1.43E-54	5.7E-53	DLL1	5.071133	2.58E-12	4.89E-11
MCL1	2.470131	2.31E-51	8.44E-50	DDIT4	4.638898	9.91E-12	1.75E-10
MET	11.26069	1.43E-48	4.73E-47	MUC1	4.301074	1.71E-11	2.91E-10
FSCN1	6.014127	6.07E-47	1.89E-45	L1CAM	3.722584	2.21E-11	3.71E-10
TNFAIP3	4.76437	1.34E-46	4.11E-45	STAT5A	6.955505	7.49E-11	1.19E-09
TP63	11.00487	3.26E-44	9.04E-43	DUSP6	1.557224	8.34E-10	1.16E-08
NOTCH2	1.479564	9.69E-44	2.63E-42	IKZF2	3.042021	1.47E-09	1.99E-08
RAB1A	1.928561	4.84E-42	1.21E-40	ERBB3	2.265969	2.13E-09	2.82E-08
HMGA2	10.71462	1.9E-41	4.65E-40	SHROOM2	2.984449	4.79E-09	6.03E-08
TIMP3	9.953337	3.5E-40	8.24E-39	NOTCH1	2.819589	5.21E-09	6.52E-08
IKZF2	5.192899	5.79E-38	1.25E-36	IGF1R	2.41391	1.2E-08	1.43E-07
ITGB8	9.726902	8.17E-38	1.75E-36	ERBB2	1.656501	2.23E-08	2.57E-07
MMP2	9.701391	1.44E-37	3.06E-36	MCL1	1.634262	2.76E-07	2.67E-06
IRS1	2.004455	1.37E-32	2.38E-31	ADAM17	1.251827	2.86E-07	2.75E-06
SOX9	5.010745	1.77E-32	3.04E-31	KLF5	1.347851	4.58E-07	4.26E-06
AXL	8.410465	2.26E-31	3.66E-30	MDM2	1.970244	4.89E-07	4.52E-06
CDC42	2.483095	5.97E-30	8.95E-29	PER1	2.023664	7.61E-06	5.64E-05
SRGAP1	2.476042	3.75E-29	5.44E-28	MFSD1	3.962113	1.12E-05	7.99E-05
FOSL1	8.730778	4.47E-29	6.45E-28	BCL2L2	1.81349	1.19E-05	8.5E-05

SOD2	1.756923	3.32E-28	4.61E-27	DUSP10	2.069389	2.15E-05	0.000145
COL4A1	8.380982	9.41E-28	1.28E-26	TPM1	1.678562	3.57E-05	0.00023
HPGD	2.971122	1.83E-25	2.22E-24	ITGA6	1.844219	3.97E-05	0.000254
MICAL2	4.753644	3.79E-25	4.51E-24	PPP2R2A	1.330882	0.000222	0.001176
DDIT4	1.642716	2.84E-24	3.22E-23	NRAS	1.502914	0.000333	0.001681
RPS6KA1	1.448549	7.13E-24	7.87E-23	PRDM1	2.338897	0.000401	0.001961
GRN	2.481532	7.6E-23	7.98E-22	SERPINE1	1.74347	0.000792	0.003563
HOXA9	7.575309	1.51E-22	1.55E-21	MAP3K9	1.379357	0.001364	0.005752
KLF12	7.94246	2.65E-22	2.69E-21	ESR1	3.454686	0.002396	0.009342
GNAI3	1.629895	2.86E-21	2.74E-20	MXD1	1.428344	0.002556	0.00986
FGFR2	5.068426	4.64E-21	4.38E-20	CYP24A1	2.891982	0.003384	0.012591
STAT1	1.302056	2.72E-20	2.47E-19	MMP1	2.249509	0.004504	0.01604
LIPA	1.950039	9.21E-19	7.68E-18	NEDD9	1.579318	0.005741	0.019756
MMP1	8.749504	1.87E-18	1.52E-17	VDR	1.241639	0.00652	0.021965
RTKN	1.523199	2.7E-18	2.16E-17	ADAMTS6	1.447687	0.00655	0.022043
FOS	6.578145	2.9E-18	2.32E-17	SMAD7	1.655764	0.006856	0.022867
MXD1	2.177361	4.45E-17	3.29E-16	MYO6	1.472437	0.007603	0.024902
TPM1	1.431471	4.11E-16	2.86E-15	VEGFA	1.257589	0.009531	0.030038
MAP3K9	2.42381	4.16E-16	2.89E-15	BBC3	1.794102	0.015462	0.044804
APAF1	2.127165	1.07E-15	7.23E-15	MFSD1	1.274416	0.020126	0.055643
L1CAM	8.61924	2.46E-15	1.62E-14	ROBO2	2.061041	0.139751	0.254676
SPRY2	3.924136	5.28E-14	3.19E-13	L1CAM	1.241457	0.306163	0.452907
ARF4	1.511863	5.74E-14	3.45E-13	HNF4A	1.2583	0.453413	NA
MTAP	1.310394	2.14E-13	1.24E-12	SHROOM2	1.81136	0.280631	NA
TCF7	3.530696	3.78E-13	2.16E-12	CDKN1A	3.753737	NA	NA
SWAP70	1.862844	1.13E-12	6.21E-12	GAS1	1.849938	0.236601	NA
BTG2	1.699401	1.18E-12	6.44E-12				
SOX2	5.682976	4.56E-11	2.16E-10				
BAK1	1.82553	5.19E-11	2.45E-10				
PRDM1	3.500605	1.17E-10	5.39E-10				
MUC1	6.045027	1.25E-10	5.72E-10				
MSH2	2.079052	2.58E-10	1.14E-09				
VDR	1.799286	3.62E-10	1.59E-09				
RICTOR	1.558435	7.8E-10	3.33E-09				
PER1	1.545091	2.15E-09	8.86E-09				
NOTCH1	1.752205	3.14E-09	1.28E-08				
ADAMTS6	3.378835	4.38E-09	1.75E-08				
PCTP	1.554791	7.95E-09	3.11E-08				
DUSP10	1.845003	4.84E-08	1.77E-07				
RB1	1.204679	5.63E-08	2.05E-07				

YES1	1.301196	1.72E-06	5.39E-06
EIF5A2	1.398772	0.000124	0.000311
CYP24A1	4.414196	0.000159	0.000392
FLI1	4.047192	0.000207	0.000501
NEDD9	1.636026	0.000325	0.000766
COL3A1	2.907007	0.004391	0.008629
ESR1	3.297547	0.011259	0.020435
DNMT3B	1.904237	0.022561	0.038526
IL12A	2.328211	0.053321	0.08411
ROBO2	1.784338	0.268362	NA

FC: Fold Change
