# Supplementary material for: Fibronectin Modulates the Expression of miRNAs in Prostate Cancer Cell Lines
Source: Front Vet Sci. 2022 Jul 11;9:879997. doi: 10.3389/fvets.2022.879997 (PMC9310065; doi:10.3389/fvets.2022.879997)
Supplement: Supplementary Table 2 | — STRING Interactions in LNCaP and PC-3 cells. [file Table_2.pdf]

LNCaP			*PC-3*		
**Node1**	**Node2**	**Combined Score**	**Node1**	**Node2**	**Combined Score**
CCND2	CDK6	0.993	MDM2	TP53	0.999
CDK6	CCND2	0.993	TP53	MDM2	0.999
BAK1	MCL1	0.992	CDKN1A	TP53	0.996
MCL1	BAK1	0.992	TP53	CDKN1A	0.996
JAG1	NOTCH1	0.991	ERBB2	ERBB3	0.991
NOTCH1	JAG1	0.991	ERBB3	ERBB2	0.991
CCND2	CDKN1A	0.99	CCND2	CDKN1A	0.99
CDK6	RB1	0.99	CDKN1A	CCND2	0.99
CDKN1A	CCND2	0.99	BBC3	MCL1	0.987
RB1	CDK6	0.99	MCL1	BBC3	0.987
CDK6	CDKN1A	0.989	PTEN	TP53	0.984
CDKN1A	CDK6	0.989	TP53	PTEN	0.984
CCND2	RB1	0.981	MDM2	TP63	0.983
RB1	CCND2	0.981	TP63	MDM2	0.983
JAG1	NOTCH2	0.978	ESR1	IGF1R	0.976
NOTCH2	JAG1	0.978	IGF1R	ESR1	0.976
APAF1	BAK1	0.951	DLL1	NOTCH1	0.953
BAK1	APAF1	0.951	NOTCH1	DLL1	0.953
MMP2	TIMP3	0.949	IGF1R	MDM2	0.95
TIMP3	MMP2	0.949	MDM2	IGF1R	0.95
HMGA2	RB1	0.948	TP53	TP63	0.95
RB1	HMGA2	0.948	TP63	TP53	0.95
FOS	SMAD3	0.947	ESR1	STAT5A	0.949
SMAD3	FOS	0.947	MMP2	TIMP3	0.949
SMAD3	VDR	0.947	STAT5A	ESR1	0.949
VDR	SMAD3	0.947	TIMP3	MMP2	0.949
ESR1	SMAD3	0.945	BBC3	TP53	0.947
SMAD3	ESR1	0.945	TP53	BBC3	0.947

ESR1	FOS	0.939	CD44	ERBB2	0.945
FOS	ESR1	0.939	ERBB2	CD44	0.945

Supp. Table II. Continued

LNCaP			*PC-3*		
**Node1**	**Node2**	**Combined Score**	**Node1**	**Node2**	**Combined Score**
CDKN1A	RB1	0.935	ESR1	FOS	0.939
RB1	CDKN1A	0.935	FOS	ESR1	0.939
CDC42	GNAI3	0.934	ERBB2	NRAS	0.934
GNAI3	CDC42	0.934	ERBB3	NRAS	0.934
ADAM17	JAG1	0.932	NRAS	ERBB3	0.934
JAG1	ADAM17	0.932	NRAS	ERBB2	0.934
CDC42	SRGAP1	0.929	FGFR2	NRAS	0.93
SRGAP1	CDC42	0.929	NRAS	FGFR2	0.93
ADAM17	NOTCH1	0.928	ADAM17	NOTCH1	0.928
NOTCH1	ADAM17	0.928	NOTCH1	ADAM17	0.928
SMAD3	TGFBR2	0.927	MCL1	TP53	0.927
TGFBR2	SMAD3	0.927	TP53	MCL1	0.927
ESR1	GNAI3	0.925	ITGA6	LAMC2	0.916
GNAI3	ESR1	0.925	LAMC2	ITGA6	0.916
HMGA2	SMAD3	0.924	CCND2	STAT5A	0.914
SMAD3	HMGA2	0.924	STAT5A	CCND2	0.914
CDKN1A	SMAD3	0.923	ADAM17	DLL1	0.911
SMAD3	CDKN1A	0.923	DLL1	ADAM17	0.911
CDC42	MET	0.922	MMP1	MMP2	0.911
MET	CDC42	0.922	MMP2	MMP1	0.911
CDC42	MAP3K9	0.921	FOS	SOX2	0.908
MAP3K9	CDC42	0.921	SOX2	FOS	0.908
ETS1	FOS	0.919	FOS	STAT5A	0.903
FOS	ETS1	0.919	SERPINE1	SMAD7	0.903
ITGA6	LAMC2	0.916	SMAD7	SERPINE1	0.903
LAMC2	ITGA6	0.916	STAT5A	FOS	0.903
FGFR2	SPRY2	0.914	BTG2	TP53	0.902

ITGA6	MET	0.914	ERBB3	ITGA6	0.902

Supp. Table II. Continued

LNCaP			*PC-3*		
**Node1**	**Node2**	**Combined Score**	**Node1**	**Node2**	**Combined Score**
MET	ITGA6	0.914	ITGA6	ERBB3	0.902
SPRY2	FGFR2	0.914	MDM2	PTEN	0.902
CDC42	YES1	0.911	MMP1	VEGFA	0.902
EPAS1	SOX2	0.911	MMP2	VEGFA	0.902
MMP1	MMP2	0.911	PTEN	MDM2	0.902
MMP2	MMP1	0.911	TP53	BTG2	0.902
SOX2	EPAS1	0.911	VEGFA	MMP2	0.902
YES1	CDC42	0.911	VEGFA	MMP1	0.902
LAMC2	MET	0.91	CDKN1A	MCL1	0.901
MET	LAMC2	0.91	MCL1	CDKN1A	0.901
FOS	SOX2	0.908	ADAM17	ERBB2	0.9
SOX2	FOS	0.908	BBC3	TP63	0.9
FGFR2	STAT1	0.907	CDKN1A	STAT5A	0.9
STAT1	FGFR2	0.907	DDIT4	TP63	0.9
MCL1	STAT1	0.905	DDIT4	TP53	0.9
STAT1	MCL1	0.905	ERBB2	ADAM17	0.9
SERPINE1	SMAD3	0.904	ERBB3	LAMC2	0.9
SMAD3	SERPINE1	0.904	FGFR2	FOS	0.9
CDC42	LAMC2	0.902	FOS	NOTCH1	0.9
FOS	FOSL1	0.902	FOS	FGFR2	0.9
FOSL1	FOS	0.902	LAMC2	MMP2	0.9
LAMC2	CDC42	0.902	LAMC2	ERBB3	0.9
CDKN1A	STAT1	0.901	MDM2	MYO6	0.9
CDKN1A	MCL1	0.901	MMP2	LAMC2	0.9
MCL1	CDKN1A	0.901	MYO6	MDM2	0.9
STAT1	CDKN1A	0.901	NOTCH1	FOS	0.9
CD44	CDC42	0.9	PRDM1	TP53	0.9
CDC42	CD44	0.9	STAT5A	CDKN1A	0.9

CDKN1A	FSCN1	0.9	TIMP3	TP53	0.9
DDIT4	TP63	0.9	TP53	TIMP3	0.9

Supp. Table II. Continued

LNCaP			*PC-3*		
**Node1**	**Node2**	**Combined Score**	**Node1**	**Node2**	**Combined Score**
FGFR2	FOS	0.9	TP53	PRDM1	0.9
FOS	MSH2	0.9	TP53	DDIT4	0.9
FOS	NOTCH1	0.9	TP63	BBC3	0.9
FOS	FGFR2	0.9	TP63	DDIT4	0.9
FSCN1	STAT1	0.9			
FSCN1	MCL1	0.9			
FSCN1	CDKN1A	0.9			
IL12A	STAT1	0.9			
LAMC2	MMP2	0.9			
MCL1	FSCN1	0.9			
MMP2	LAMC2	0.9			
MSH2	FOS	0.9			
NOTCH1	FOS	0.9			
STAT1	IL12A	0.9			
STAT1	FSCN1	0.9			
TP63	DDIT4	0.9			
